# Serum cytokines and creatinine/cystatin C ratio as prognostic biomarkers in advanced cancer patients treated with anti-PD-1/PD-L1 therapy

**DOI:** 10.1007/s00520-024-08525-z

**Published:** 2024-05-22

**Authors:** Shan-xiu Jin, Bo-Na Liu, Hong-juan Ji, Jing-ran Wu, Bao-lei Li, Xiao-li Gao, Na Li, Zhen-dong Zheng, Cheng Du

**Affiliations:** 1 Department of Oncology, General Hospital of Northern Theater Command, Shenyang, China; 2 Department of Oncology, Anshan Tumor Hospital, Anshan, China; 3 Department of Gynaecology and Obstetrics, The First Hospital of Jilin University, Jilin, China

**Keywords:** Serum cytokines, Creatinine/cystatin C ratio, Prognostic biomarkers, Cancer, Immune checkpoint inhibitors

## Abstract

**Objective:**

Immune checkpoint inhibitors (ICIs), specifically targeting the programmed cell death protein-1 or its ligand (PD-1/PD-L1), have been extensively used in the treatment of a spectrum of malignancies, although the predictive biomarkers remain to be elucidated. This study aims to investigate the association between baseline circulating levels of cytokines and the creatinine/cystatin C ratio (CCR) with the treatment outcomes of ICIs in patients with advanced cancer.

**Methods:**

The pre-treatment circulating levels of 10 cytokines (PD-L1, CTLA4, CXCL10, LAG3, HGF, CCL2, MIG, GRANB, IL-18, and IL-6) were measured via automated capillary-based immunoassay platform in the serum of 65 advanced cancer patients treated with anti-PD-1/PD-L1-based systemic therapy and 10 healthy volunteers. The levels of cytokines and CCR were quantified and categorized into high and low groups based on the median value. The associations of serum cytokines and CCR with response to treatment, survival, and immune-related adverse events were assessed.

**Results:**

Elevated circulating levels of 6 cytokines (PD-L1, CXCL10, HGF, CCL2, MIG, and IL-6) were observed in cancer patients compared with that in healthy volunteers. The correlation coefficients between cytokines, CCR and nutritional risk index were also calculated. In the cancer cohort (*N* = 65), low circulating HGF (*P* = 0.023, *P* = 0.029), low IL-6 (*P* = 0.002, *P* < 0.001), and high CCR (*P* = 0.031, *P* = 0.008) were associated with significantly improved progression-free survival (PFS) and overall survival (OS). Multi-variable COX analyses adjusted for clinicopathological factors revealed that low HGF, low IL-6, and high CCR were independent favorable prognostic factors for PFS (*P* = 0.028, *P* = 0.010, and *P* = 0.015, respectively) and OS (*P* = 0.043, *P* = 0.003, and *P* = 0.026, respectively). Grade 2 irAEs occurred more frequently in patients with low levels of circulating CCL2 and LAG3.

**Conclusions:**

Pre-treatment circulating levels of serum IL-6, HGF, and CCR may serve as independent predictive and prognostic biomarkers in advanced cancer patients treated with ICIs-based systemic therapy. These findings might help to identify potential patients who would benefit from these therapies.

**Supplementary Information:**

The online version contains supplementary material available at 10.1007/s00520-024-08525-z.

## Introduction

It is estimated that 19.3 million new cancer cases and almost 10.0 million cancer deaths occurred in 2020 worldwide [1]. Immune checkpoint inhibitors (ICIs) specifically targeting PD-1 or PD-L1, have greatly improved the prognosis of cancer patients and emerged as the standard of care for a broad spectrum of malignancies [2]. Despite these advancements, the elevated response rate and prolonged survival is still limited in non-selected patients [3–5]. This may be due to genetic tumor heterogeneity, the non-immunogenic tumor microenvironment, and the impaired nutritional status and immune capacity of individuals [6, 7]. Therefore, identifying predicative and prognostic biomarkers for cancer patients undergoing ICIs-based treatment is crucial and remains to be investigated.

Cytokines are proteins secreted by both immune cells and non-immune cells (such as endothelial cells, epidermal cells, and fibroblasts). They have a wide range of biological activities. Cytokines are associated with the diagnosis and prognosis of various cancerous disease. Among the cytokines, interleukin is usually associated with inflammation [8]. Previous studies have uncovered that some cytokines, such as interleukin 6, 15, and 18(IL-6, IL-15, and IL-18), in peripheral blood are associated with the treatment outcomes of ICIs in NSCLC [9–11]. Hepatocyte growth factor/c-mesenchymal epithelial transition factor (HGF/c-Met) signaling mediates the crosstalk between immune-cell and tumor micro-environment [12]. It is suggested that HGF may be candidate of ICI biomarkers. Circulating cytokines, such as soluble immune checkpoints, can modulate immune response to cancer cells. However, the studies of many circulating cytokines are still unclear in cancer patients receiving ICIs therapy. It is important to find appropriate cytokines to predict the prognosis of patients with cancer treated with ICIs-based systemic therapy.

Serum creatinine (Cr) and cystatin C (Cys C) are commonly utilized to assess renal function in clinical practice. Creatinine is mainly derived from the metabolism of muscles. Cys C is not affected through muscular metabolic processes [13]. Therefore, leveraging the characteristics of these two markers ratio, the serum creatinine/cystatin C ratio (CCR) has been served as an alternative biomarker of sarcopenia and prognostic factor in cancer patients. However, whether CCR is associated with the efficacy of ICIs-based treatment remains unclear.

In this study, we investigated the association of circulating levels of 10 cytokines and CCR with the outcomes of advanced cancer patients treated with ICIs-based systemic therapy.

## Materials and methods

### Patient characteristics

From June 2021 to December 2021, 65 cancer patients at the General Hospital of Northern Theater Command were enrolled in the study. Additionally, 10 healthy controls, displaying good health without any indications of tumors, were included. The inclusion criteria for the cancer patients were as follows: histopathological confirmation of cancer at advanced stage or cannot be surgically resected, receiving anti-PD-1/PD-L1 inhibitors-based system therapies, the Eastern Cooperative Oncology Group Performances Status (ECOG) ranging from 0 to 2 informed consent written from patients for the collection of blood samples and clinical information. This study was approved by the institutional ethics board of General Hospital of Northern Theater Command and was performed adhering to the principles outlined in the Declaration of Helsinki by World Medical Association.

### Response assessment and nutritional index

The clinical response was categorized into four groups: complete response (CR), partial response (PR), stable disease (SD), or progressive disease (PD). Evaluation of the response to ICIs treatment was conducted according to the response evaluation criteria in solid tumors version 1.1 (RECIST 1.1). Durable clinical benefit (DCB) was defined as achieving a partial response or stable disease with a PFS duration of ≥ 6 months. Non-durable benefit (NDB) encompassed other response categories. PFS and OS were documented based on medical records or follow-up phone calls. Two indexes reflecting the nutritional status of patients were calculated as follows: Nutritional risk index (NRI) = [1.519 × albumin concentration (g/L)] + [41.7 × (current weight/standard weight)]. CCR = creatinine (mg/dL)/ cystatin C (mg/L) × 100.

### Sample collection and cytokine detection

Peripheral blood was collected before the initiation of anti-PD-1/PD-L1 therapy and centrifuged at 1000 g at room temperature for 15 min. Subsequently, 1 mL of serum was promptly transferred into a 1.5 mL centrifuge tube and cryopreserved at -80℃ until analysis. We extracted 100 μL of serum from a previously frozen centrifuge tube for each sample. Serum samples were centrifuged at high speed (12000rpm) at room temperature for 3 min. Then, supernate was diluted by the certain and appropriate amount and was shaken on a plate shaker (approximately 400–500 rpm) for 2 h at 25 ℃.Finally, add 1 mL of wash buffer and 50 μL of the diluted sample to each well of the test panel. For cytokine quantification, a customized panel was employed, enabling the simultaneous assessment of multiple cytokines. This analysis was conducted on an automated capillary-based immunoassay platform (ProteinSimple, Ella-21050762) provided by Bio-Techne in China.

### Statistical analyses

Independent t-tests were utilized to analyze differences of cytokines and CCR in patients grouped by clinicopathological variables. Mann–Whitney or independent t-tests are used to compare the difference of cytokine between patients and healthy volunteers. Spearman correlation analysis was employed to determine correlations between serum factors. Kaplan–Meier curves for OS and PFS were presented according to the median values. The impact of different blood biomarker levels on OS and PFS was estimated using a Cox regression analysis. Receiver operating characteristic (ROC) curve analysis was used to evaluate the utility of the cytokines for predicting 6, 12, and 18 months of OS. The data of irAEs was presented as categorical variables and analyzed using the chi-squared test and Fisher’s exact test. All P values were two-sided, with a significance threshold set at *P* < 0.05. SPSS (version 26) and R (version 4.1.2) were used for statistical analyses.

## Results

### Patient characteristics and comparison of cytokine levels between cancer and control groups

**Fig. 1:**
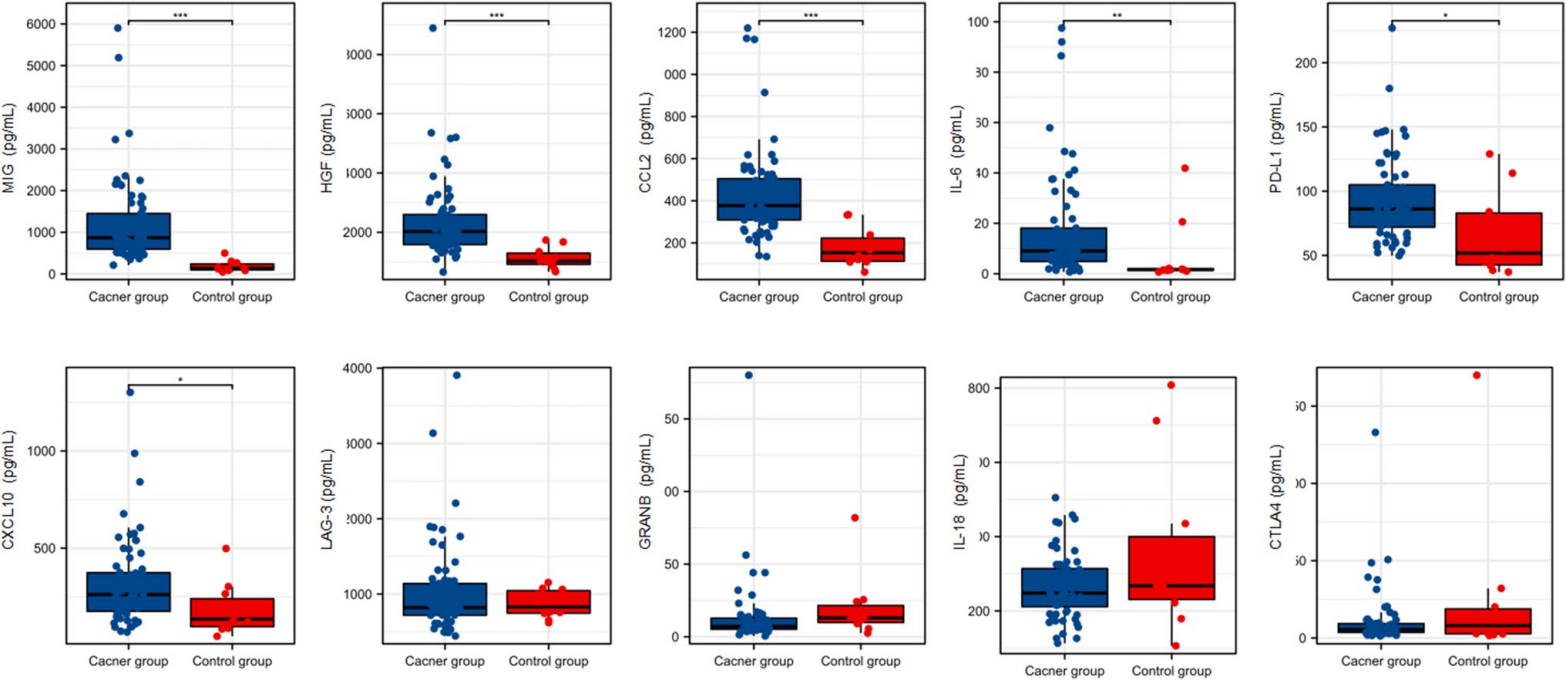
The difference between cancer (*n* = 65) and healthy group (*n* = 10) in terms of cytokines. The line in the middle of the box shows the mean value. Error bars show the interquartile range. (MIG: *P* < 0.001, HGF: *P* < 0.001, CCL2: *P* < 0.001, IL-6: *P* = 0.03, PD-L1: *P* = 0.012, CXCL10: *P* = 0.022, LAG-3: *P* = 0.761, GRANB: *P* = 0.068, IL-18: *P* = 0.462, CTLA4: *P* = 0.9441)

The clinicalpathological characteristics of the 65 patients at baseline were summarized in Table 1. The male displayed higher circulating levels of IL-6, mitogen-inducible gene (MIG) and CCR than female. No significant differences in cytokine levels were observed in terms of smoking or drinking history. The predominant cancer types were lung cancer [22(33.8%)] and gastroesophageal cancer [16(24.6%)]. 34 (52.3%) patients were administered PD-1 plus chemotherapy, while 14 patients (21.5%) received PD-L1 plus chemotherapy. Of note, 20 (54.1%) patients displayed positive expression of PD-L1 and exhibited elevated levels of CCL2. Tumor response to ICIs therapy, assessed using RECIST 1.1 criteria, showed an objective response rate (ORR) of 30.7% and a disease control rate (DCR) of 87.1%. No significant differences in terms of cytokines were observed between different treatment response groups. There was no difference in age (*P* = 0.177) and sex (*P* = 0.062) between the cancer and control groups. The levels of circulating PD-L1, CXCL10, MIG, HGF, CCL-2, and IL-6 were significantly higher in the cancer group compared with those of the control group. (*P* = 0.012, *P* = 0.022, *P* < 0.001, *P* < 0.001, *P* < 0.001, and *P* = 0.030, respectively) (Fig. 1).

**Table 1:** Clinicalpathological characteristics of the patients (mean ± sd)

Variables		PD-L1 (pg/ml)	CTLA-4 (pg/ml)	CXCL-10 (pg/ml)	MIG (pg/ml)	HGF (pg/ml)	IL-18 (pg/ml)	LAG-3 (pg/ml)	CCL2 (pg/ml)	GRANB (pg/ml)	IL-6 (pg/ml)	CCR	NRI
Sex	*n*												
Male	50	95.9 ± 33.1	12.1 ± 20.2	322.1 ± 230.6	1314.6 ± 1145.2	2375.0 ± 1342.3	267.9 ± 85.4	1011.2 ± 632.8	447.4 ± 221.2	14.8 ± 26.3	19.7 ± 23.3	70.4 ± 13.7	100.0 ± 9.5
Female	15	85.2 ± 31.2	7.7 ± 11.4	296.5 ± 195.4	934.5 ± 379.5	2275.9 ± 1165.4	255.1 ± 84.8	1084.7 ± 470.4	381.2 ± 195.1	8.2 ± 4.5	9.4 ± 8.2	61.1 ± 16.1	103.5 ± 9.1
*P* value		0.274	0.427	0.699	0.049	0.797	0.610	0.679	0.301	0.337	0.010	0.029	0.212
Age													
≤ 65	31	97.6 ± 38.5	9.4 ± 8.6	354.6 ± 264.4	1444.7 ± 1304.8	2442.1 ± 1559.9	268.6 ± 78.8	1131.2 ± 767.9	444.2 ± 223.8	17.3 ± 32.4	21.2 ± 24.3	68.8 ± 15.8	101.0 ± 9.7
> 65	34	89.6 ± 26.5	12.6 ± 24.5	281.1 ± 170.8	1028.3 ± 652.7	2270.1 ± 1014.2	261.7 ± 90.9	934.2 ± 367.8	421.1 ± 211.3	9.6 ± 8.2	13.9 ± 17.5	67.7 ± 13.8	100.7 ± 9.3
*P* value		0.328	0.493	0.184	0.116	0.597	0.746	0.201	0.670	0.203	0.167	0.766	0.875
Smoking													
Yes	36	90.9 ± 26.8	7.9 ± 8.1	285.9 ± 206.3	1138.1 ± 885.2	2355.0 ± 1044.7	270.0 ± 83.2	1016.0 ± 708.0	415.6 ± 197.2	15.4 ± 30.5	20.0 ± 22.8	70.1 ± 14.2	101.3 ± 9.8
No	29	96.5 ± 39.3	15..0 ± 26.1	353.8 ± 237.9	1337.1 ± 1193.5	2348.6 ± 1572.3	258.7 ± 87.7	1043.2 ± 432.0	452.7 ± 239.1	10.6 ± 8.3	14.0 ± 18.9	65.9 ± 15.2	100.3 ± 9.1
*P* value		0.500	0.169	0.223	0.443	0.984	0.598	0.857	0.495	0.411	0.259	0.249	0.663
Drinking													
Yes	28	90.4 ± 29.5	8.1 ± 8.6	281.1 ± 197.3	1157.4 ± 1014.6	2275.0 ± 1006.5	266.2 ± 80.9	891.8 ± 349.9	398.5 ± 189.2	9.6 ± 7.3	20.6 ± 24.4	69.9 ± 15.9	101.3 ± 11.2
No	37	95.6 ± 35.3	13.4 ± 23.4	342.7 ± 237.8	1279.4 ± 1053.0	2410.5 ± 1488.6	264.1 ± 88.7	1131.3 ± 718.1	457.6 ± 233.4	16.1 ± 30.1	14.9 ± 18.3	67.0 ± 13.7	100.5 ± 7.9
*P* value		0.528	0.265	0.271	0.640	0.680	0.921	0.082	0.278	0.272	0.281	0.434	0.728
ECOG													
< 2	58	91.9 ± 32.3	11.6 ± 19.6	322.8 ± 228.0	1262.7 ± 1072.3	2264.3 ± 1268.1	267.0 ± 84.8	1047.9 ± 613.8	443.3 ± 224.8	13.2 ± 23.9	15.7 ± 20.1	69.4 ± 14.6	102.0 ± 8.5
= 2	7	105.9 ± 36.7	6.6 ± 6.3	261.2 ± 163.7	930.0 ± 535.2	3080.0 ± 1390.4	248.4 ± 89.0	864.0 ± 428.0	339.7 ± 77.8	14.3 ± 18.7	30.6 ± 26.7	58.8 ± 12.4	91.5 ± 11.9
*P* value		0.288	0.506	0.492	0.424	0.116	0.589	0.445	0.233	0.904	0.079	0.072	0.005
Diagnose													
Non-small cell lung cancer	22	86.5 ± 30.2	5.6 ± 4.0	226.4 ± 124.2	874.5 ± 489.3	2135.9 ± 952.2	262.4 ± 76.4	831.4 ± 344.7	351.2 ± 218.8	9.2 ± 11.1	19.4 ± 25.8	68.1 ± 14.7	104.0 ± 11.3
Gastroesophageal cancer	16	87.7 ± 22.7	9.9 ± 11.7	321.4 ± 137.1	1548.6 ± 1229.4	2097.5 ± 766.3	233.1 ± 52.1	867.8 ± 230.0	472.1 ± 207.0	8.1 ± 3.6	16.5 ± 16.5	69.9 ± 15.4	98.5 ± 6.5
Other	27	102.4 ± 38.3	16.2 ± 26.6	386.2 ± 292.9	1323.3 ± 1170.3	2679.3 ± 1694.0	285.9 ± 101.6	1283.4 ± 796.0	474.4 ± 207.1	19.7 ± 34.0	16.2 ± 20.2	67.4 ± 14.7	99.6 ± 8.9
*P* value		0.178	0.070	0.018	0.112	0.296	0.079	0.035	0.095	0.220	0.856	0.859	0.135
Therapy													
PD-1 plus targeted therapy	9	112.3 ± 37.3	26.0 ± 41.1	528.6 ± 371.8	1704.4 ± 1679.9	2452.2 ± 1467.1	237.7 ± 95.1	1455.3 ± 1125.2	417.4 ± 148.2	15.8 ± 17.4	12.1 ± 11.0	68.5 ± 20.0	97.0 ± 5.2
PD-1 plus chemotherapy	34	89.7 ± 33.4	9.3 ± 11.5	268.9 ± 153.6	1199.1 ± 992.8	2283.7 ± 1481.0	271.0 ± 76.8	849.9 ± 251.5	404.5 ± 225.5	12.9 ± 29.9	21.4 ± 27.9	69.4 ± 14.9	102.4 ± 10.2
PD-L1 plus chemotherapy	14	88.2 ± 24.6	8.1 ± 12.0	254.4 ± 192.8	908.1 ± 469.4	2258.5 ± 751.1	262.6 ± 107.4	1136.1 ± 711.1	459.07 ± 173.4	11.9 ± 11.3	12.5 ± 5.0	67.6 ± 12.6	101.0 ± 11.3
PD-1	8	96.8 ± 35.3	7.2 ± 4.2	386.4 ± 170.7	1365.6 ± 950.2	2694.2 ± 1145.1	274.1 ± 69.6	1116.2 ± 386.5	518.9 ± 302.5	14.4 ± 12.6	14.2 ± 9.3	64.5 ± 12.3	98.2 ± 4.2
*P* value		0.281	0.516	0.114	0.334	0.861	0.759	0.114	0.560	0.981	0.382	0.865	0.165
PD-L1 status													
Negative	17	91.6 ± 17.9	12.5 ± 14.2	306.8 ± 201.2	1000.6 ± 577.7	2775.4 ± 1225.6	251.4 ± 77.4	1103.0 ± 835.5	482.1 ± 179.8	12.4 ± 11.4	21.3 ± 24.3	70.5 ± 14.7	101.8 ± 8.6
Positive	20	83.8 ± 29.8	7.1 ± 5.4	267.7 ± 180.8	1142.3 ± 748.5	2138.2 ± 943.5	259.0 ± 88.9	886.8 ± 329.3	355.0 ± 93.8	21.0 ± 40.0	10.4 ± 9.4	69.0 ± 13.6	101.8 ± 9.8
unknown	28												
*P* value		0.351	0.156	0.538	0.529	0.083	0.785	0.294	0.009	0.396	0.096	0.751	0.995
Response to treatment	*n*												
PD	8	114.5 ± 52.7	8.7 ± 6.7	398.0 ± 387.6	1541.8 ± 1819.9	3574.1 ± 2362.4	278.6 ± 111.1	1164.0 ± 585.9	451.9 ± 109.1	16.2 ± 16.5	31.8 ± 28.2	61.6 ± 13.6	95.7 ± 11.7
SD	35	89.5 ± 29.3	12.0 ± 23.3	319.1 ± 190.5	1265.0 ± 953.6	2161.3 ± 1115.9	258.4 ± 80.4	1127.0 ± 720.4	433.4 ± 260.2	12.9 ± 29.7	12.0 ± 13.9	67.3 ± 15.5	100.6 ± 8.0
PR	19	95.7 ± 27.2	11.2 ± 13.3	290.1 ± 206.6	1132.3 ± 795.0	2275.8 ± 791.2	274.5 ± 89.7	838.16 ± 241.9	414.5 ± 167.2	11.6 ± 9.7	22.4 ± 27.1	70.4 ± 12.3	102.5 ± 11.0
unknown	3												
*P* value		0.151	0.906	0.534	0.655	0.292	0.741	0.073	0.914	0.901	0.096	0.352	0.252

*sd* Standard deviation; *PD* Progressive Disease; *SD* Stable Disease; *PR* Partial Response; *CPS* Combined Positive Score; *ECOG* Eastern Cooperative Oncology Group Performance Status; *CCR* Creatinine/cystatin C ratio; *NRI* Nutritional risk index. *P* < 0.05 was considered statistically significant and shown in bold type

### Correlation analysis of cytokines and nutritional indexes

**Fig. 2:**
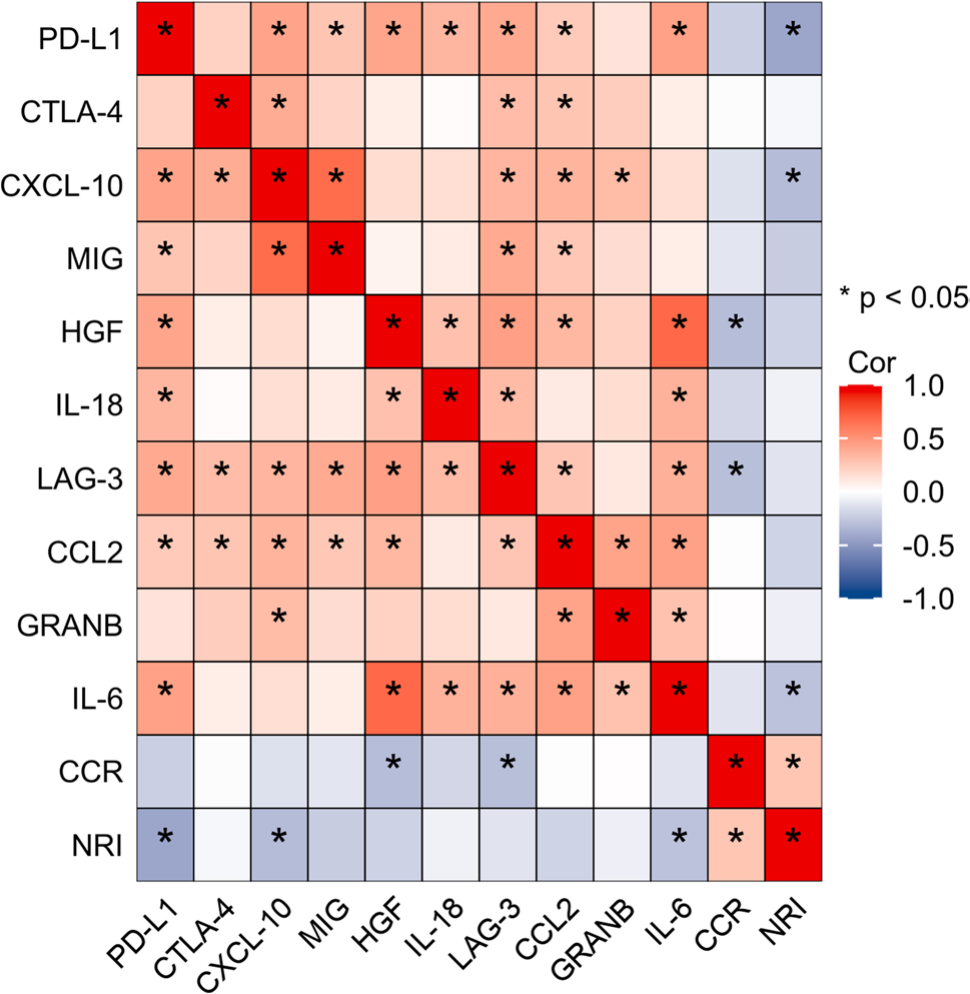
The heatmap showing the correlation between cytokines, CCR, and NRI. The color shows the degree of correlation

A heat-map of the correlation between the soluble cytokines and nutritional indexes were presented in Fig. 2. It’s essential to highlight that the correlation coefficient of CCR with NRI is 0.27 (*P* = 0.030). The circulating level of CCR was negatively correlated with that of LAG-3 (R = -0.303, *P* = 0.014) and HGF (R = -0.311, *P* = 0.012). Likewise, NRI was negatively correlated with PD-L1 (R = -0.423, *P* < 0.001), IL-6 (R = -0.288, *P* = 0.02), and CXCL10 (R = -0.306, *P* = 0.013).LAG-3 was positively correlated with PD-L1 (R = 0.407, *P* < 0.001), CTLA4 (R = 0.371, *P* = 0.001), MIG (R = 0.402, *P* < 0.001), IL-18 (R = 0.321, *P* = 0.009), CXCL10 (R = 0.349, *P* = 0.004), CCL2 (R = 0.272, *P* = 0.028), IL-6 (R = 0.371, *P* = 0.003) and HGF (R = 0.294, *P* = 0.018). CCL2 was positively correlated with PD-L1 (R = 0.251, *P* = 0.044), CTLA4 (R = 0.274, *P* = 0.027), MIG (R = 0.264, *P* = 0.034), HGF (R = 0.337, *P* = 0.006), CXCL10 (R = 0.354, *P* = 0.004), IL-6 (R = 0.444, *P* < 0.001) and GRANB (R = 0.435, *P* < 0.001). The correlation coefficient of HGF and IL-6 is 0.687.The detail of correlation analysis of cytokines and CCR was shown in Supplementary Table S1 and Figure S1.

### Associations between cytokines and clinical benefits

We compared serum cytokines and CCR concentrations in DCB and NCB groups. The NCB group had elevated serum concentrations of HGF (2365 pg/mL vs. 1769 pg/mL, *P* = 0.006) and IL-6(16.6 pg/mL vs. 7.195 pg/mL, *P* = 0.001) compared with the DCB group (Table 2). Univariate analysis showed that low HGF and low IL-6 were significant prognostic factors for DCB. Multivariate logistics regression analysis revealed that the low level of IL-6 tends to independently predict DCB (*P* = 0.062, Table 3). The highest AUC (0.743) for DCB was observed in patients with IL-6 alone.The AUCs for DCB in patients of either IL-6^low^ and/or HGF^low^ levels (*n* = 39), and IL-6^low^ and HGF^low^ (*n* = 25) were 0.729 and 0.629, respectively, which were lower than the AUC of IL-6 alone. (Supplementary Figure S2).

**Table 2:** Differences of cytokines in patients between NCB and DCB groups

	NCB	DCB	*P* value
*n*	23	42	
PD-L1, median (IQR)	89.8 (71.5, 109.0)	84.3 (72.5, 102.1)	0.676
CTLA-4, median (IQR)	5.1 (3.4, 9.1)	5.6 (3.7, 9.2)	0.597
CXCL-10, median (IQR)	250.0 (184.0, 443.5)	262 (173.5, 367.3)	0.773
MIG, median (IQR)	778 (523.5, 1184.5)	932.0 (680.8, 1669.5)	0.219
HGF, median (IQR)	2365.0 (1876.5, 3278.0)	1769.0 (1557.8, 2309.2)	0.006
IL-18, mean ± sd	265.7 ± 88.0	264.4 ± 85.0	0.954
LAG-3, median (IQR)	874 (741.5, 1180.5)	804.5 (718.5, 1125.5)	0.403
CCL2, median (IQR)	454 (311.5, 555.5)	372.5 (303.8, 447.8)	0.227
GRANB, median (IQR)	8.4 (6.3, 14.0)	6.52 (4.8, 11.2)	0.110
IL-6, median (IQR)	16.6 (9.1, 34.6)	7.195 (3.7, 10.8)	0.001
CCR, mean ± sd	65.6 ± 15.8	69.7 ± 14.0	0.280
NRI, mean ± sd	99.6 ± 11.2	101.5 ± 8.4	0.465

*NCB* Non-durable clinical benefit; *DCB* Durable clinical benefit; *IQR* Inter-quartile range; *CCR* Creatinine/cystatin C ratio; *NRI* Nutritional risk index

**Table 3:** Univariate and multivariate logistic regression analysis for NBC/DBC

Characteristics	Total(*N*)	Univariate analysis		Multivariate analysis	
		Odds Ratio (95% CI)	*P* value	Odds Ratio (95% CI)	*P* value
Sex	65				
Male	50	Reference		Reference	
Female	15	1.125 (0.332—3.807)	0.850	1.769 (0.319—9.808)	0.514
Age	65				
≤ 65	31	Reference		Reference	
> 65	34	0.769 (0.277—2.139)	0.615	0.676 (0.205—2.232)	0.520
Smoking history	65				
Yes	36	Reference		Reference	
No	29	1.074 (0.386—2.990)	0.891	1.261 (0.270—5.879)	0.768
Alcohol history	65				
Yes	28	Reference		Reference	
No	37	0.438 (0.149—1.281)	0.132	0.336 (0.076—1.493)	0.152
Diagnose	65				
Other	27	Reference		Reference	
Gastroesophageal cancer	16	2.062 (0.525—8.096)	0.299	1.420 (0.285—7.072)	0.668
Non-small cell lung cancer	22	1.203 (0.377—3.835)	0.755	1.292 (0.318—5.251)	0.720
HGF	65				
High	33	Reference		Reference	
Low	32	3.361 (1.140—9.908)	0.028	1.628 (0.402—6.588)	0.494
IL-6	65				
High	33	Reference		Reference	
Low	32	6.480 (2.002—20.978)	0.002	3.993 (0.933—17.100)	0.062

*ECOG* Eastern Cooperative Oncology Group Performance Status; *CCR* Creatinine/cystatin C ratio; *NRI* Nutritional risk index; *CI* Confidence interval

### Prognostic value of cytokines and nutritional indexes

**Fig. 3:**
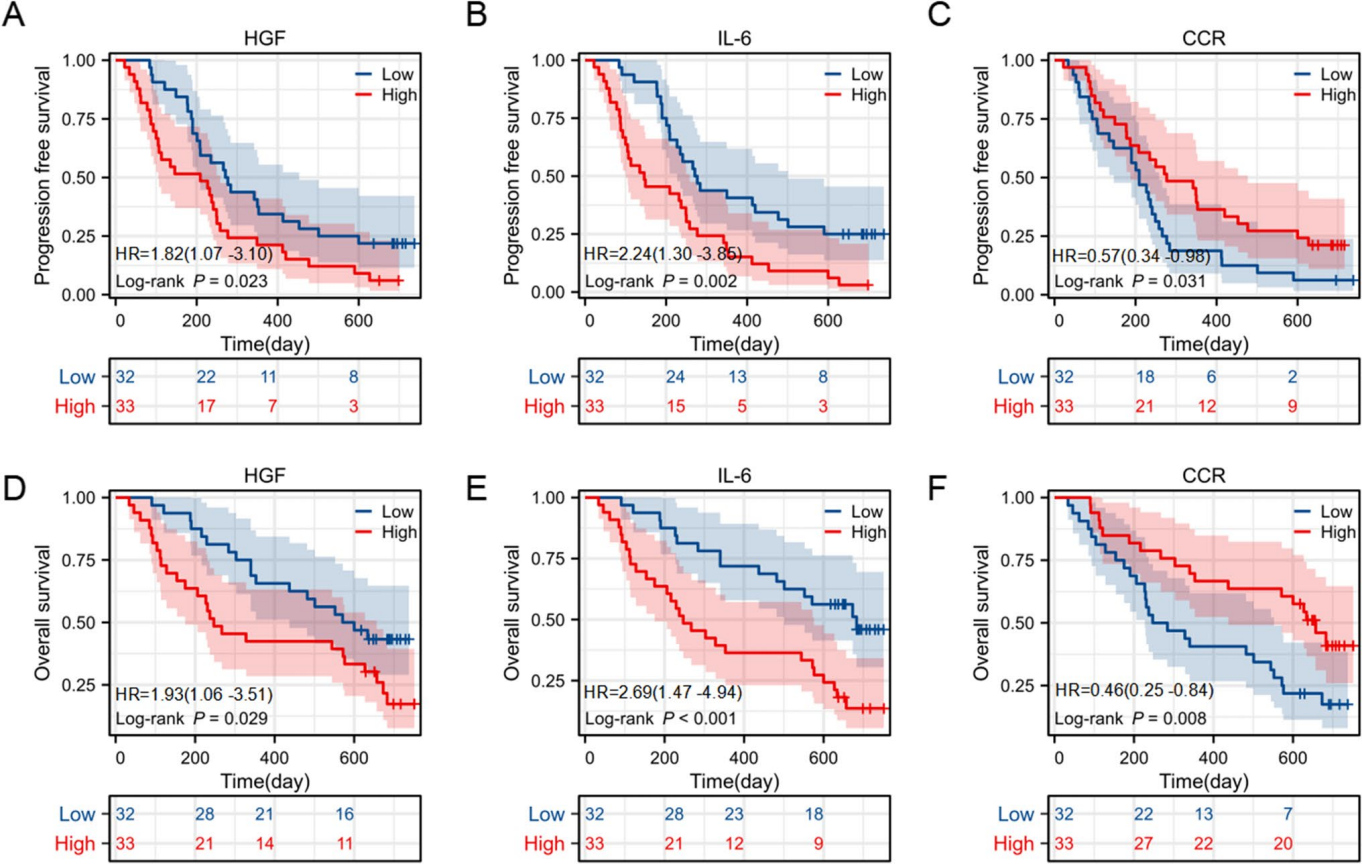
Kaplan–Meier curve for PFS and OS between patients with high HGF **A**, **D**, IL6 **B**, **E**, or CCR **C**, **F** and those with low HGF, IL6, or CCR

**Fig. 4:**
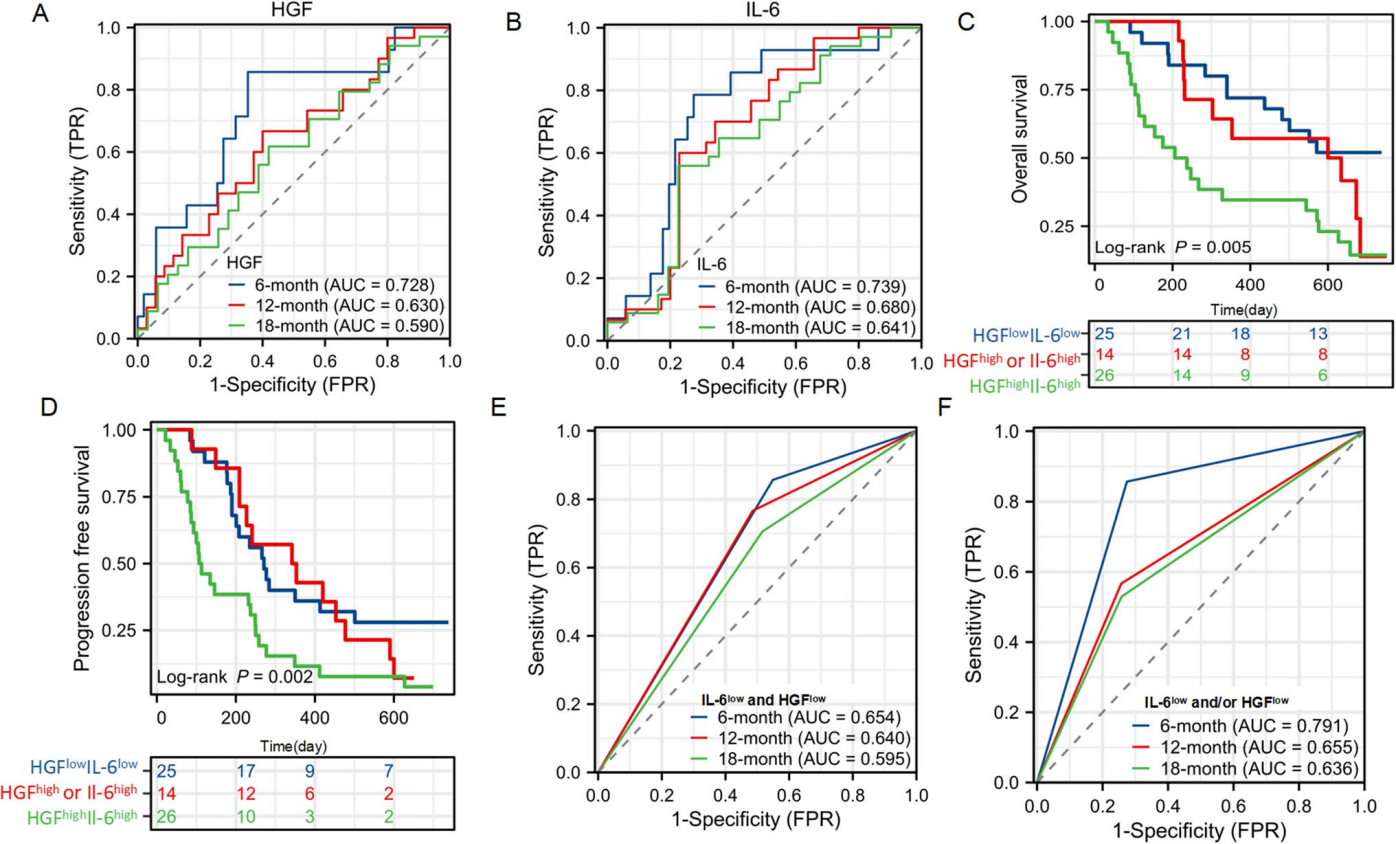
ROC curves of HGF **A** and IL6 **B** predicting 6, 12, and 18 months survival. Kaplan–Meier curves for OS **C** and PFS **D** on the basis of HGF and IL-6 levels classified as both low, either high, or both high. **E** ROC curves of IL-6^low^ and HGF^low^ predicting 6, 12, and 18 months survival. **F** ROC curves of IL-6^low^ and HGF^low^ predicting 6, 12, and 18 months survival

We next tested whether survival curves for PFS and OS were stratified by the levels of the 10 cytokines and CCR using the median values as cut-offs. Higher levels of circulating IL-6 and HGF were significantly associated with shorter PFS and OS (*P* = 0.002 and *P* < 0.001 for IL-6, *P* = 0.023 and *P* = 0.029 for HGF). Patients with CCR levels above the median showed longer PFS and OS (*P* = 0.031 and *P* = 0.008 respectively, Fig. 3). Furthermore, we performed ROC analysis to determine the utility of HGF and IL-6 in predicting survival. HGF was determined at cut-off values of 6-month, 12-month, and 18-month OS, and yielded AUCs of 0.728, 0.630, and 0.590 respectively, indicating that the 6- month OS cutoff possessed greater predictive value. These cutoffs were compared with IL-6, which generated AUCs of 0.739, 0.680, and 0.641 at 6-month, 12-month, and 18-month OS cutoffs, respectively. These results demonstrated that IL-6 was superior to HGF in predicting survival (Fig. 4A, B). Notably, to further explore the predictive abilities of these biomarkers, we combined the patients into the following three groups: both IL-6 and HGF low group; either IL-6 or HGF high group; and both IL-6 and HGF high group. Survival curves for PFS and OS were clearly stratified into the two distinct groups, with the worst PFS and OS in the IL-6 and HGF high group (*P* = 0.002 and *P* = 0.005, Fig. 4C, D). The AUCs for 6-month, 12-month, and 18-month OS in patients with IL-6^low^ and HGF^low^ levels were 0.654, 0.640 and 0.595, respectively. The AUCs for 6-month, 12-month, and 18-month OS in patients with IL-6^low^ and/or HGF^low^ levels were 0.791, 0.655 and 0.636, respectively (Fig. 4E, F). Finally, we analyzed the data using the Cox proportional hazards model with known risk factors for OS and PFS. As shown in Table 4, HGF IL-6 and CCR were shown to be possibly related to OS and PFS in the univariate analysis. When adjusting for HGF, IL-6 or CCR alone, all were independent prognostic factors (PFS: *P* = 0.028, *P* = 0.010, and *P* = 0.016, respectively, OS: *P* = 0.043, *P* = 0.003, and *P* = 0.026, respectively, Tables 5 and 6). However, when adjusting for 3 factors simultaneously including HGF, IL6, and CCR, only IL-6 and CCR were independent factors for prognosis, indicating that the prognostic effect of HGF could be obscured by IL-6 and CCR (Supplement Table S2).

**Table 4:** Univariate COX regression analysis for PFS and OS

Characteristics	Total(*N*)	PFS		OS	
		Hazard ratio (95% CI)	*P* value	Hazard ratio (95% CI)	*P* value
Sex	65				
Male	50	Reference		Reference	
Female	15	1.277 (0.695—2.346)	0.432	1.647 (0.843—3.220)	0.144
Age	65	1.008 (0.979—1.038)	0.580	0.998 (0.964—1.033)	0.912
Smoking history	65				
Yes	29	Reference		Reference	
No	36	1.034 (0.608—1.757)	0.901	1.063 (0.583—1.936)	0.842
Alcohol history	65				
Yes	37	Reference		Reference	
No	28	0.980 (0.578—1.663)	0.941	1.264 (0.692—2.309)	0.446
Diagnose	65				
Other	27	Reference		Reference	
Gastroesophageal cancer	16	0.747 (0.376—1.486)	0.406	0.648 (0.302—1.389)	0.264
Non-small cell lung cancer	22	1.145 (0.628—2.089)	0.658	0.753 (0.380—1.492)	0.416
ECOG	65				
< 2	58	Reference		Reference	
= 2	7	2.975 (1.326—6.678)	0.008	2.544 (1.069—6.052)	0.035
HGF	65				
Low	33	Reference		Reference	
High	32	1.831 (1.078—3.111)	0.025	1.934 (1.059—3.533)	0.032
IL-6	65				
Low	33	Reference		Reference	
High	32	2.286 (1.337—3.908)	0.003	2.746 (1.477—5.107)	0.001
CCR	65				
Low	33	Reference		Reference	
High	32	0.559 (0.328—0.954)	0.033	0.451 (0.245—0.828)	0.010

*ECOG* Eastern Cooperative Oncology Group Performance Status; *CCR* Creatinine/cystatin C ratio; *NRI* nutritional risk index; *CI* Confidence interval

**Table 5:** Multivariate COX regression analysis for PFS

Characteristics	Total(*N*)	HGF model		IL-6 model		CCR model	
		Hazard ratio (95% CI)	*P* value	Hazard ratio (95% CI)	*P* value	Hazard ratio (95% CI)	*P* value
Sex	65						
Male	50	Reference		Reference		Reference	
Female	15	0.985 (0.457—2.121)	0.969	1.156 (0.530—2.519)	0.716	0.685 (0.301—1.558)	0.367
Age	65	1.001 (0.966—1.037)	0.955	1.006 (0.970—1.043)	0.745	0.996 (0.962—1.032)	0.841
Smoking history	65						
Yes	36	Reference		Reference		Reference	
No	29	1.083 (0.501—2.340)	0.839	1.128 (0.513—2.481)	0.765	0.784 (0.341—1.806)	0.568
Alcohol history	65						
Yes	28	Reference		Reference		Reference	
No	37	0.970 (0.469—2.006)	0.934	0.878 (0.418—1.845)	0.732	1.116 (0.522—2.385)	0.778
Diagnose	65						
Non-small cell lung carcinoma	22	Reference		Reference		Reference	
Gastrointestinal cancer	17	0.614 (0.278—1.356)	0.227	0.629 (0.285—1.391)	0.252	0.585 (0.258—1.327)	0.200
Others	26	0.887 (0.440—1.786)	0.737	0.941 (0.487—1.819)	0.856	0.935 (0.468—1.869)	0.849
ECOG	65						
< 2	58	Reference		Reference		Reference	
= 2	7	3.184 (1.316—7.702)	0.010	2.093 (0.829—5.283)	0.118	3.500 (1.448—8.458)	0.005
HGF	65						
Low	32	Reference					
High	33	1.866 (1.071—3.257)	0.028				
IL-6	65						
Low	32			Reference			
High	33			2.179 (1.209 -3.922)	0.010		
CCR	65						
Low	32					Reference	
High	33					0.430 (0.218—0.849)	0.015

HGF model: adjusting for sex, age, smoking history, alcohol history, diagnose, ECOG and HGF

IL-6 model: adjusting for sex, age, smoking history, alcohol history, diagnose, ECOG and IL-6

CCR model: adjusting for sex, age, smoking history, alcohol history, diagnose, ECOG and CCR

*ECOG* Eastern Cooperative Oncology Group Performance Status; *CCR* Creatinine/cystatin C ratio; *NRI* Nutritional risk index; *CI* Confidence interval

**Table 6:** Multivariate COX regression analysis for OS

Characteristics	Total(*N*)	HGF model		IL-6 model		CCR model	
		Hazard ratio (95% CI)	*P* value	Hazard ratio (95% CI)	*P* value	Hazard ratio (95% CI)	*P* value
Sex	65						
Male	50	Reference		Reference		Reference	
Female	15	1.639 (0.712—3.773)	0.245	1.980 (0.833—4.707)	0.122	1.210 (0.505—2.901)	0.669
Age	65	0.993 (0.954—1.034)	0.739	0.991 (0.951—1.033)	0.676	0.985 (0.946—1.026)	0.468
Smoking history	65						
Yes	36	Reference		Reference		Reference	
No	29	0.907 (0.393—2.090)	0.818	1.031 (0.438—2.427)	0.945	0.690 (0.289—1.644)	0.402
Alcohol history	65						
Yes	28	Reference		Reference		Reference	
No	37	1.160 (0.509—2.644)	0.723	1.071 (0.462—2.485)	0.873	1.320 (0.569—3.063)	0.518
Diagnose	65						
Non-small cell lung carcinoma	22	Reference		Reference		Reference	
Gastrointestinal cancer	17	0.763 (0.313—1.859)	0.552	0.879 (0.361—2.141)	0.777	0.764 (0.307—1.906)	0.564
Others	26	0.599 (0.260—1.378)	0.228	0.676 (0.312—1.465)	0.321	0.663 (0.290—1.511)	0.328
ECOG	65						
< 2	58	Reference		Reference		Reference	
= 2	7	2.562 (0.993—6.612)	0.052	1.764 (0.660—4.714)	0.257	2.577 (0.990—6.710)	0.053
HGF	65						
Low	32	Reference					
High	33	1.946(1.022—3.704)	0.043				
IL-6	65						
Low	32			Reference			
High	33			2.899 (1.449 -5.814)	0.003		
CCR	65						
Low	32					Reference	
High	33					0.447 (0.220—0.907)	0.026

HGF model: adjusting for sex, age, smoking history, alcohol history, diagnose, ECOG and HGF

IL-6 model: adjusting for sex, age, smoking history, alcohol history, diagnose, ECOG and IL-6

CCR model: adjusting for sex, age, smoking history, alcohol history, diagnose, ECOG and CCR

*ECOG* Eastern Cooperative Oncology Group Performance Status; *CCR* Creatinine/cystatin C ratio; *NRI* Nutritional risk index; *CI* Confidence interval

### Associations between cytokines and irAEs

**Fig. 5:**
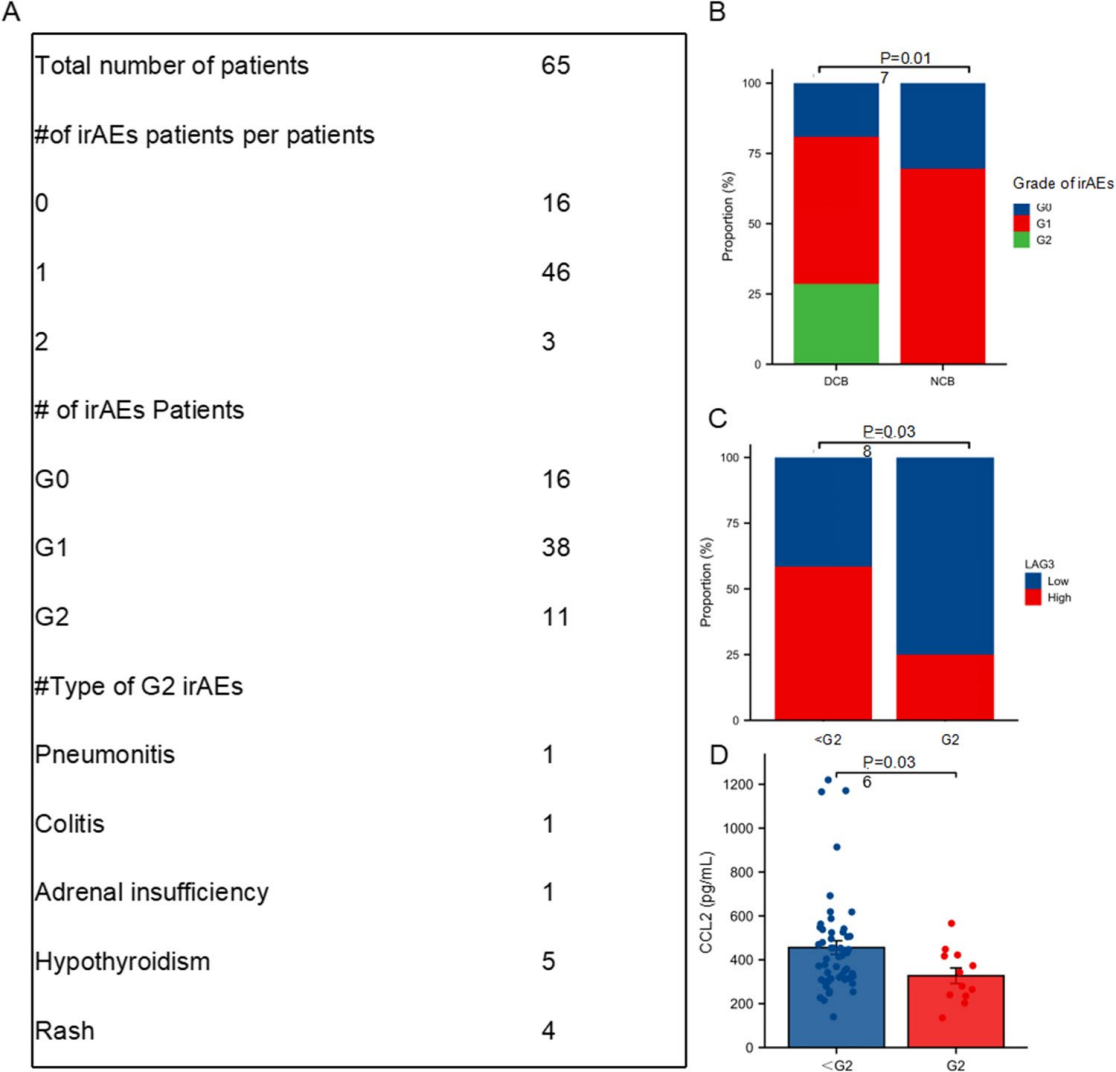
Cytokines in relation to irAEs. **A** The frequency, severity, and type of irAEs. **B** Proportion of patients who experienced different grade of irAEs on the basis of DCB and NCB. **C** Proportion of patients with high or low level LAG3 on the basis of grade of irAEs. **D** Comparisons of serum CCL2 levels according to the grade of irAEs

We next explored cytokines in relation to clinical appearance of irAEs. At the time of analysis, 11 of these patients were identified as having grade 2 irAEs. Patients in DCB group were more prone to occur irAEs (Fig. 5A, B). Low level of lymphocyte activation gene-3 (LAG3) was correlated with the occurrence of irAEs (Fig. 5C). Patients experiencing grade 2 irAEs also expressed high levels of C–C motif chemokine ligand 2 (CCL2) than patients with grade 1 irAEs (Fig. 5D).

## Discussion

In this study, we demonstrated significantly higher levels of circulating PD-L1, CXCL10, MIG, HGF, CCL-2, and IL-6 in cancer patients compared to normal healthy subjects. In cancer patients, low circulating HGF, low IL-6, and high CCR were associated with improved PFS and OS. Moreover, pre-treatment levels of circulating IL-6, HGF, and CCR may serve as independent predictive and prognostic biomarkers in advanced cancer patients. Finally, low levels of CCL2 and LAG3 were correlated with the occurrence of irAEs.

As a pro-inflammatory cytokine implicated in chronic inflammatory conditions, IL-6 is reported to affect the efficacy of immunotherapy [14, 15]. The potential mechanism involves the IL-6-STAT3 signaling pathway, which attenuates the cytotoxic effects of cluster of differentiation 8 positive (CD8 +) T cells and leads to the down-regulation of the expression of major histocompatibility complex class II on the surface of dendritic cells [16–18]. Some recent studies have also shown an association between high baseline IL-6 levels and reduced benefits from immunotherapy in hepatocellular carcinoma, melanoma, renal cell carcinoma, lung cancer and cutaneous squamous cell carcinoma patients [14, 19–22]. These studies support our findings that high level of IL-6 indicates a poor prognosis for advanced cancer patients treated with ICIs. Targeting IL-6 and ICIs could be a new therapeutic strategy [23].

HGF is secreted by mesenchymal cells and acts as a multi-functional cytokine mainly on epithelial cells. HGF binds to the proto-oncogenic c-Met receptor, activating tyrosine kinase signaling cascades and regulating cell growth, cell motility, and apoptosis [24]. The neutrophils could be recruited into T cell-inflamed environments through HGF/c-MET [25]. Therefore, the activated HGF/c-MET pathway is considered as a negative regulator for cancer immunotherapy [26, 27]. Conversely, inhibition of c-MET was reported to activate the anti-cancer immune response by promoting the cytotoxic effects of CD8 + T cells [28]. A recent study showed that low level of circulating HGF correlated to superior response to anti-PD-1 therapy in patients with metastatic melanoma [29]. And another study demonstrated that lower baseline plasma HGF are potential biomarkers for anti-angiogenesis therapy and immunotherapy in advanced triple-negative breast cancer patients [30]. Our study also found that low level of serum HGF might be a good prognostic factor for cancer patients receiving ICIs-based systemic therapy.

Emerging evidence indicated that CCR is a promising indicator in predicting the sarcopenia [31–33]. A recent study showed that high level of pre-treatment CCR significantly correlated to improved response and survival in NSCLC patients who received PD-1 inhibitor monotherapy [34]. In another study, a lower CCR and a lower SI (sarcopenia index, SI = serum creatinine × cystatin C) are independent predictors of mortality, in metastatic non-small cell lung cancer patients treated with PD-1 inhibitors [35]. However, in our study, neither SI and sarcopenia (according to the criteria commonly referenced in Asian cancer patients [36]) was found to be predictive of prognosis, nor SI was found to be associated with SMA (Supplementary Figure S3). On the other hand, in accordance with the above mentioned two studies, we also demonstrated that high level of serum CCR indicated better outcomes in our cohort of heterogenous cancer patients receiving ICIs-based systemic therapy.

Although high-grade irAEs are rare, they can significantly impact both quality of life and treatment outcomes. A study on esophageal cancer showed that CCL2 inhibits the recruitment of tumor-associated macrophages and the polarization of type II macrophages. It stops immunosuppression against tumor effector T cells through the PD-1 signaling pathway [37]. According to a recent study, CCL2 was reported to be a potential predictor of neurotoxicity in cancer patients treated with ICIs [38]. LAG-3 is a type of immune checkpoint receptor protein, which is mainly expressed on activated T cells and negatively regulates T cell function. Recently, LAG-3 was found to be involved in the differentiation and survival of auto-reactive CD8 + T-cell [39, 40]. PD-1/LAG-3 double-positive CD8 + T cells showed enhanced cytotoxic capacity in patients receiving ICIs with irAEs [41]. Therefore, serum LAG3 might predict both therapeutic and adverse effects of ICIs in cancer patients. In this study, low levels of LAG3 and CCL2 seemed to be associated with high-grade irAEs, although their role in predicting treatment response was not proved in our data analysis, which might be explained by the limited sample size and heterogeneity of disease context.

Several limitations of this study should be acknowledged. Firstly, this study enrolled heterogeneous cancer patients treated with different regiments based on anti-PD-1/PD-L1, thus the conclusions should be interpreted with caution. Secondly, the sample size is too small to design a validation cohort, which might improve the strength of the study. Finally, we did not evaluate the expression of these cytokines or their receptors on cancer cells and immune cells, which might also be involved in regulating the response of immunotherapy. We hope to initiate a largescale study focusing on homogenous disease treated with ICIs in the future.

In conclusion, pre-treatment circulating levels of serum IL-6, HGF, and CCR may serve as independent predictive and prognostic biomarkers in advanced cancer patients treated with ICIs-based systemic therapy. These findings might help to identify potential patients who would benefits from these therapies.

### Supplementary Information

Below is the link to the electronic supplementary material.

Supplementary file1 (DOCX 895 KB)
