# Supplementary material for: Serum cytokines and creatinine/cystatin C ratio as prognostic biomarkers in advanced cancer patients treated with anti-PD-1/PD-L1 therapy
Source: Support Care Cancer. 2024 May 22;32(6):370. doi: 10.1007/s00520-024-08525-z (PMC11111483; doi:10.1007/s00520-024-08525-z)
Supplement: Supplementary file 1 — Supplementary file1 (DOCX 895 KB) [file 520_2024_8525_MOESM1_ESM.docx]

| **Cor**  **P** | PD-L1 | CTLA-4 | CXCL-10 | MIG | HGF | IL-18 | LAG-3 | CCL2 | GRANB | IL-6 | CCR | NRI |
| --- | --- | --- | --- | --- | --- | --- | --- | --- | --- | --- | --- | --- |
| PD-L1 |  | 0.213 | 0.429 | 0.275 | 0.425 | 0.330 | 0.407 | 0.251 | 0.137 | 0.442 | -0.226 | -0.423 |
| CTLA-4 | 0.088 |  | 0.393 | 0.203 | 0.081 | 0.018 | 0.317 | 0.274 | 0.235 | 0.086 | -0.013 | -0.036 |
| CXCL-10 | 0.000 | 0.001 |  | 0.671 | 0.163 | 0.160 | 0.349 | 0.354 | 0.318 | 0.158 | -0.145 | -0.315 |
| MIG | 0.026 | 0.106 | 0.000 |  | 0.055 | 0.098 | 0.402 | 0.264 | 0.166 | 0.080 | -0.117 | -0.240 |
| HGF | 0.000 | 0.521 | 0.195 | 0.664 |  | 0.294 | 0.450 | 0.337 | 0.211 | 0.687 | -0.311 | -0.209 |
| IL-18 | 0.007 | 0.885 | 0.204 | 0.436 | 0.018 |  | 0.321 | 0.092 | 0.159 | 0.358 | -0.198 | -0.065 |
| LAG-3 | 0.001 | 0.010 | 0.004 | 0.001 | 0.000 | 0.009 |  | 0.272 | 0.110 | 0.371 | -0.303 | -0.127 |
| CCL2 | 0.044 | 0.027 | 0.004 | 0.034 | 0.006 | 0.465 | 0.028 |  | 0.435 | 0.444 | -0.008 | -0.207 |
| GRANB | 0.277 | 0.060 | 0.010 | 0.187 | 0.091 | 0.206 | 0.381 | 0.000 |  | 0.291 | 0.008 | -0.073 |
| IL-6 | 0.000 | 0.496 | 0.209 | 0.524 | 0.000 | 0.003 | 0.002 | 0.000 | 0.019 |  | -0.128 | -0.288 |
| CCR | 0.071 | 0.916 | 0.248 | 0.353 | 0.012 | 0.113 | 0.014 | 0.949 | 0.949 | 0.311 |  | 0.270 |
| NRI | 0.000 | 0.774 | 0.011 | 0.054 | 0.095 | 0.608 | 0.312 | 0.099 | 0.565 | 0.020 | 0.030 |  |

**Supplementary Table S1 Association between cytokine, CCR, and NRI**

*Abbreviation: Cor: correlation coefficient, CCR: creatinine/cystatin C ratio, NRI: nutritional risk index. CI:confidence interv*

**Supplement Table S2 Multivariate COX regression analysis for HGF, IL-6, and CCR. model**

| Characteristics | Total(N) | PFS | |  | OS | |
| --- | --- | --- | --- | --- | --- | --- |
|  |  | Hazard ratio (95% CI) | P value |  | Hazard ratio (95% CI) | P value |
| **ECOG** | 65 |  |  |  |  |  |
| ＜2 | 58 | Reference |  |  | Reference |  |
| ≥2 | 7 | 2.041 (0.869 - 4.794) | 0.101 |  | 1.137 (0.444 - 2.912) | 0.790 |
| **HGF** | 65 |  |  |  |  |  |
| Low | 32 | Reference |  |  | Reference |  |
| High | 33 | 1.263 (0.681 - 2.344) | 0.458 |  | 0.944 (0.442 - 2.017) | 0.882 |
| **IL-6** | 65 |  |  |  |  |  |
| Low | 32 | Reference |  |  | Reference |  |
| High | 33 | 2.050 (1.067 - 3.938) | **0.031** |  | 2.867 (1.299 - 6.324) | **0.009** |
| **CCR** | 65 |  |  |  |  |  |
| Low | 32 | Reference |  |  | Reference |  |
| High | 33 | 0.521 (0.294 - 0.920) | **0.025** |  | 0.433 (0.223 - 0.840) | **0.013** |

*Adjusting for sex, age, smoking history, alcohol history, diagnose, ECOG, HGF, IL-6, and CCR.*

*Abbreviation: ECOG: Eastern Cooperative Oncology Group Performance Status, CCR: creatinine/cystatin C ratio, NRI: nutritional risk index. CI:confidence interv*


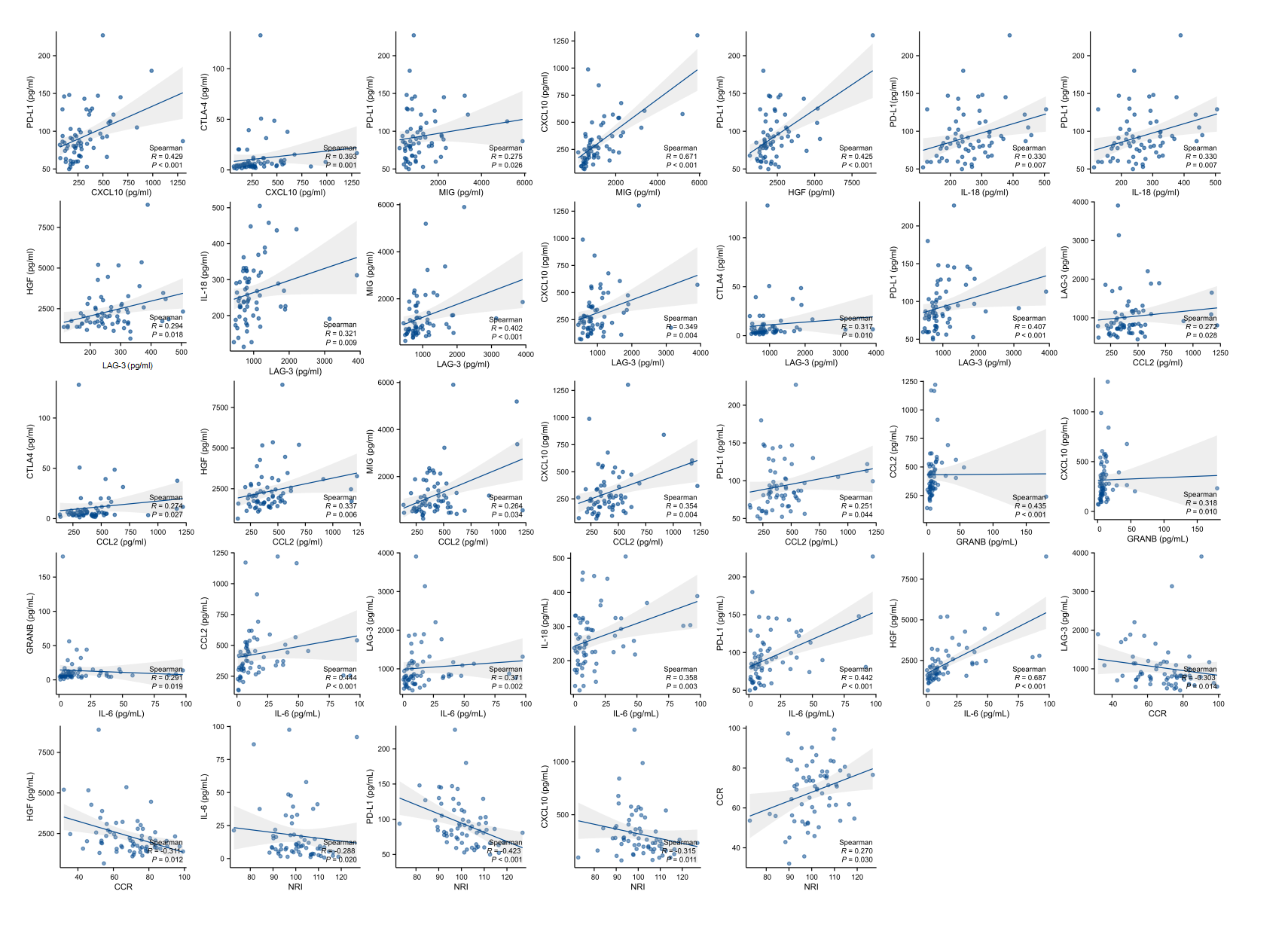


**Supplementary Figure S1** Linear correlation between cytokines NRI and CCR. (P＜0.05 is shown above)


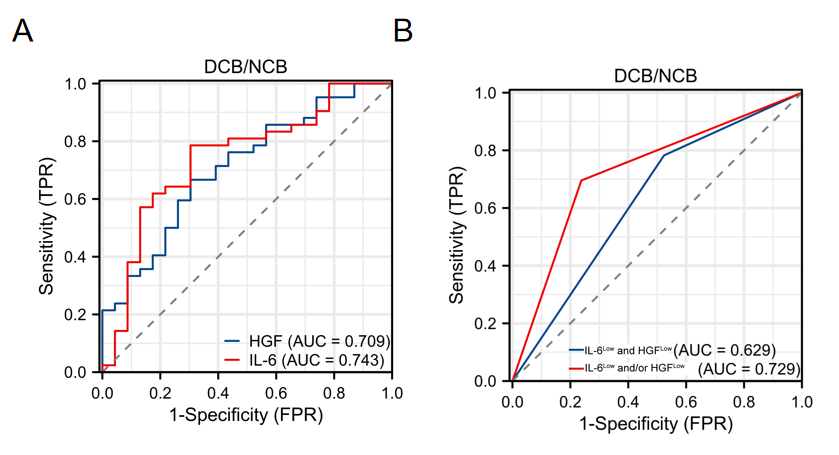


**Supplementary FigureS2** (A) The ROC curves of HGF and IL-6 for predicting DCB, (B) The ROC curves of IL-6^low^ and HGF^low^ levels and IL-6^low^ and/or HGF^low^ levels for predicting DCB


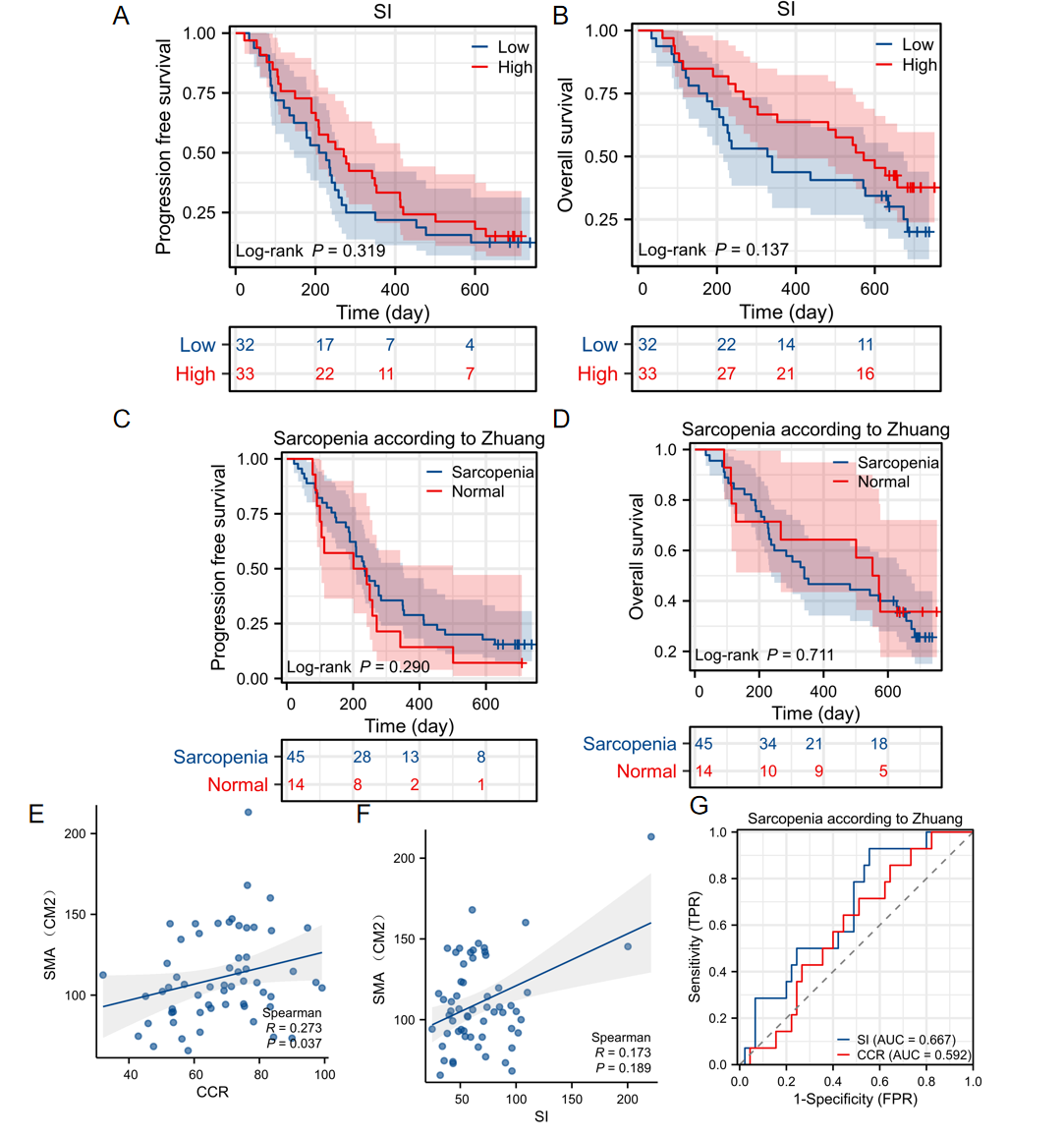


**Supplementary Figure S3** Kaplan-Meier curve for PFS and OS between patients with high SMA (A, B), and sarcopenia (C, D) and those with low HGF, IL6, or CCR Linear correlation between SMA and CCR (E) or SI (F). The ROC curve for SI and CCR predicting sarcopenia (G).
